# Proximal-type epithelioid sarcoma – a rare, aggressive subtype of epithelioid sarcoma presenting as a recurrent perineal mass in a middle- aged male

**DOI:** 10.1186/1477-7819-5-28

**Published:** 2007-03-06

**Authors:** Bharat Rekhi, Biru D Gorad, Roshni F Chinoy

**Affiliations:** 1 Department of Pathology, Tata Memorial Hospital, Parel, Mumbai, India

## Abstract

**Background:**

Epithelioid sarcoma (ES) is an uncommon soft tissue sarcoma. Lately, subtypes of ES, including proximal-type ES have been recognized, with relatively few reports on such cases.

**Case presentation:**

A 47-year-old male presented with a perineal soft tissue mass that was excised elsewhere and the biopsy was submitted for a review diagnosis. On histology, a multi nodular tumor was seen comprising sheets of oval to polygonal cells with moderate amount of cytoplasm. Interspersed were larger, rhabdoid cells with abundant eosinophilic cytoplasm and prominent nucleoli. Focal necrosis was noted. A wide panel of immunohistochemical (IHC) markers was performed to rule out a range of differential diagnoses, including a poorly differentiated carcinoma, a melanoma and a variety of sarcomas with epithelioid differentiation. On IHC, the tumor cells showed a polyphenotypic expression, including positivity for epithelial markers i.e cytokeratin (CK), CK7, EMA and mesenchymal markers like vimentin and CD 34. Desmin was focally positive. CK20, CEA, S-100, HMB-45, SMA, LCA and CD31 were negative. A diagnosis of a proximal-type ES was formed. Six moths later, despite adjuvant chemo and radiotherapy (CT and RT), the patient continued to have the lesion and was referred again. In addition to the earlier histological features, sections from the persistent tumor mass showed an increased number of larger cells along with multinucleated tumor giant cells.

**Conclusion:**

The value of identifying this uncommon tumor from a list of differential diagnoses is in view of its aggressive behavior, as seen in our case. A wide excision with clear margins is imperative with options of post-operative CT/RT in individual cases during a close follow-up.

## Background

Epithelioid sarcoma (ES) is an uncommon soft tissue sarcoma, first described as an independent entity by Enzinger [1]. This is usually a slow growing tumor and mostly occurs in the dermal or subcutaneous area of the distal extremities of young adults. It has a tendency for development of local recurrences and metastasis thereafter, which has been seen in 40–45% cases. Apart from the conventional type of ES, fibrous histiocytoma-like and angiomatoid subtypes have also been noted [2,3]. Lately, an aggressive subtype of ES has been identified known as the "proximal-type/axial-type" [4]. We present this uncommmon tumor in a middle-aged male, who presented with a perineal mass. The diagnosis is discussed in the light of available literature along with a wide range of differentials that were considered.

## Case presentation

A 47-year-old male presented with a gradually increasing, painful soft tissue mass of 7–8 months duration, measuring 4 × 3 cm in the inner side of his right thigh. A computed tomogram (CT) scan revealed an abnormal enhancing lesion in the perianal region. All the visceral organs were found to be normal. No bowel wall thickening, free fluid or abdominal lymphadenopathies were noticed.

Subsequently, he underwent a marginal excision for this mass, elsewhere. It was diagnosed as a poorly differentiated carcinoma on biopsy and referred to us in form of a single hematoxylin and eosin (H & E) stained micro section along with a paraffin block for review.

At our Hospital, his tumor marker levels for carcino embryogenic antigen (CEA) were normal i.e. 1.8 ng/ml (normal range: 0.3–2.7 ng/ml)

On review histology, a diagnosis of a "proximal-type" epithelioid sarcoma was offered. The patient was recommended a wide excision post a magnetic resonance imaging (MRI) for fear of residual disease. However, he was lost to follow-up.

Six moths later, he presented with an enlarging mass at the same location. During this time gap, he revealed a history of having undergone adjuvant chemotherapy (CT) and radiotherapy (RT) that were well tolerated. However, he developed difficulty in walking as a result of the persistent lesion. He underwent a chest X-ray along with CT scan and a real time B-mode high frequency ultrasonographic (USG) examination for the persistent mass that was re-excised and re-submitted to us for review.

### Ultrasonographic (USG) findings

**Figure 1:**
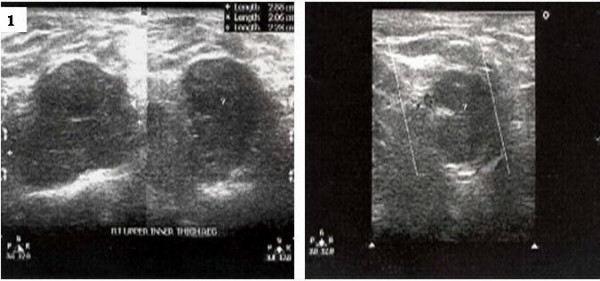
Real-time B-mode high frequency USG findings of the recurrent lesion: An oval, hypoechoic solid lesion anteriosuperior to a scar in the subcutaneous region of the right upper inner thigh.

An oval, hypoechoic, solid lesion measuring 2.9 × 2.2 × 2.1 cms was seen in the deep subcutaneous region of the right, upper, inner thigh up to 5–10 mm superior and anterior to the scar of the earlier excision. Other, two, small oval hypoechoic solid lesions of sizes 10 and 8 mm were seen in the subcutaneous layer. The main lesion showed an ill-defined echogenic wall with peripheral echogenic linear echo entering the lesion, suggestive for a lymph node. Eccentric cortical parenchyma showed a distorted architecture. The overlying skin was normal with slightly prominent subcutaneous fat lobules. No significant flow lesion was seen in the color Doppler. Radiologically, the differential diagnoses included a tumor mass vs a lymph node mass. (Figure 1).

### Histopathological findings

Grossly, the first excision specimen was in form of grey-white soft tissue bits aggregating to 3.5 × 2 × 2 cms. Details regarding the marginal status were unavailable.

### Microscopic findings

**Figure 2:**
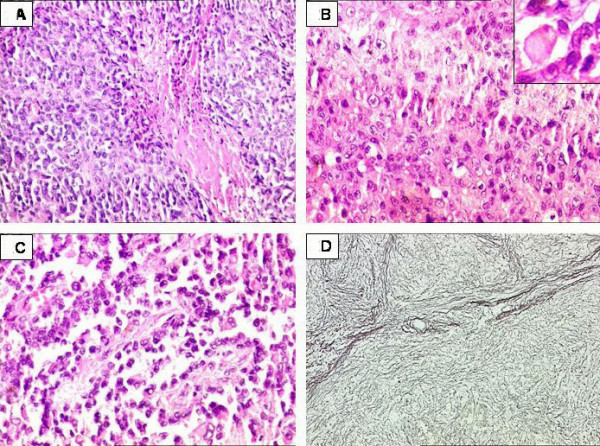
Proximal-type epithelioid sarcoma of perineum. 2A. Tumor cells showing compact sheets of polygonal cells. Focal tumoral necrosis noted. H&E × 200. 2B. Sheets of large cells with occasional intracytoplasmic inclusions (inset) and little intervening stroma. H&E × 400. 2C. A 'pseudoangiosarcomatous' pattern. Tumor cells arranged in a disintegrating perivascular pattern. H&E × 400. 2D. Prominent multi nodular pattern of tumor. Reticulin silver stain × 100.

On histology, the tumor revealed a multi nodular pattern as a result of investing thin fibrous septa with cells predominantly arranged in a cohesive manner. At places cellular disintegration was observed in combination with hemorrhage resulting in a 'pseudoangiosarcomatous' pattern. The cells were oval to polygonal and exhibited moderate nuclear pleomorphism. Interspersed were larger cells with vesicular nuclei, prominent nucleoli and abundant cytoplasm, including intracytoplasmic inclusions reminiscent of a 'rhabdoid' morphology along with focal areas of tumoral necrosis. Mitoses were noted, however, not conspicuously. (Figure 2A, 2B, and 2C).

**Figure 3:**
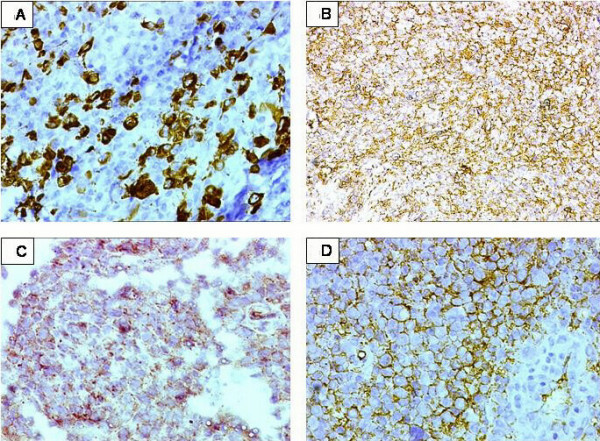
IHC results. 3A. Tumor cells displaying positive immunoreactivity for CK7. DAB × 400. 3B. Tumor cells displaying strong membranous positivity for EMA. DAB × 200. 3C. Tumor cells showing intra cytoplasmic dot-like expression for desmin. DAB × 400. 3D. Strong, diffuse, membranous positivity for CD34. DAB × 400. Positive internal control in the vascular endothelial cells noted.

Special histochemical stains were carried out with a wide panel of IHC markers (Table 1). The reticulin staining highlighted the nodular growth pattern of the tumor. (Figure 2D). IHC showed a diffuse strong positivity for CK and vimentin in the tumor cells. CK-7 and EMA were also strongly positive. (Figure 3A and 3B). Desmin was focally positive (Figure 3C). In addition, CD34 was found to be strongly positive (Figure 3D). SMA, Myo-D1, CK-20, CEA, S-100, HMB-45, CD 31, C-kit and LCA were negative. A diagnosis of a proximal-type epithelioid sarcoma of was formed.

**Table 1:** List of the various primary antibody markers

**Antigen**	**Type**	**Dilution**	**Antigen retrieval**	**Source**
Vimentin	Monoclonal	1:50	Microwave	Dako, Produkionsveg, Glostrup, Denmark
Cytokeratin (CK)	Monoclonal	1:100	Pronase (Enzymatic)	Dako
CK 7	Monoclonal	1:50	Microwave	Dako
Epithelial membrane antigen (EMA)	Monoclonal	1:100	Pepsin (Enzymatic)	Dako
Desmin	Monoclonal	1:50	Microwave	Dako
CD34	Monoclonal	1:100	Microwave	Dako
Smooth muscle actin (SMA)	Monoclonal	1:200	Pepsin (Enzymatic)	Dako
CK20	Monoclonal	1:100	Microwave	Dako
Carcinoembryonic antigen (CEA)	Polyclonal	1:400	Pepsin (Enzymatic)	Dako
S-100	Polyclonal	1:300	Pepsin (Enzymatic)	Dako
Myo D-1	Monoclonal	1:20	Pressure cooker and EDTA	Dako
HMB-45	Monoclonal	1:50	Microwave	Dako
Leukocyte common antigen (LCA)	Monoclonal	1:100	Microwave	Dako
C-KIT	Polyclonal	1:100	Microwave	Dako
Calretinin	Monoclonal	1:50	Microwave	Dako
CD31	Monoclonal	1:40	Microwave	Dako

The re-excision showed a similar histology. In addition, increased number of larger cells was noticed along with tumor giant cells and several mitoses.

## Discussion

A conventional-type epithelioid sarcoma (ES) it is mostly seen in the distal extremities (commonly hand) of young adults. On histomorphology, it displays a typical "granuloma-like" pattern of tumor cells surrounding a necrotic focus [1].

Lately, a non-conventional subtype of ES has been identified with a differing clinical profile and a polyphenotypic differentiation, termed as the "proximal-type" ES. It is more frequently seen in the pelvis, perineum and genital tract of young to middle aged adults. On histology, it usually lacks the conventional "granuloma-like appearance". In addition to presence of oval to polygonal cells, it shows characteristic large cells with 'rhabdoid' traits [4].

The present case was identified as a perineal mass in a middle-aged male. The outside slide on review revealed a multi nodular tumor comprising oval to polygonal cells arranged predominantly in cohesive sheet like pattern. Interspersed were large cells with abundant cytoplasm, vesicular nuclear chromatin and prominent nucleoli. A wide panel of IHC makers was performed to sort out a diagnosis from an equally wide range of differentials, including metastasis from a poorly differentiated carcinoma, a rhabdomyosarcoma, a melanoma, an epithelioid leiomyosarcoma, an epithelioid angiosarcoma, an epithelioid malignant peripheral nerve sheath tumor (MPNST), an epithelioid gastrointestinal stromal tumor (GIST), a conventional ES, an anaplastic large cell lymphoma (ALCL), a synovial sarcoma, an extra-renal rhabdoid tumor and a mesothelioma. In the clinico-radiologic metastatic work-up, no definite mass was identified in the solid organs as well as in the gastrointestinal (GI) tract. In addition, CEA levels were found to be normal. No glandular or squamoid areas were seen in the tumor. Further, on IHC, lack of CK20 and CEA expression made a possibility of a metastatic adenocarcinoma from the GI tract as less likely. CD 34 positivity further substantiated a diagnosis of an ES over a carcinoma, as well as a synovial sarcoma, the latter which is known to express epithelial and mesenchymal markers [5,6]. Apart from this dual immunoreactivity, no biphasic tumor cell pattern was noted. Lack of S-100 and HMB-45 ruled out the possibilities of an epithelioid MPNST and a melanoma. Desmin was found to be focally positive as has been seen by Guillou et al in 62.5% of their cases of proximal-type ES [4]. Possibility of an epithelioid rhabdomyosarcoma was discarded considering strong immunoreactvity for CK, CK7 and EMA along with Myo D-1 negativity. An epithelioid leiomyosarcoma was ruled out in view of SMA negativity. An epithelioid GIST seemed another differential, but lack of C-KIT was helpful in considering this close differential diagnosis, less likely.

An interesting observation in the tumor was a 'pseudoangiosarcomatous' pattern, as has been noted in some cases by Guillou et al [4]. Lack of CD31 ruled out the possibility of an epithelioid angiosarcoma [7]. In addition, diffuse EMA and CK expression helped in considering a diagnosis of an epithelioid angiosarcomas as less likely, which can rarely show focal expression for these markers [5]. Lack of LCA along with diffuse positivity for CK ruled out an ALCL. Apart from the non-contributory findings in terms of any body cavity lesions, calretinin negativity ruled out a mesothelioma. In this way, a diagnosis of proximal-type ES was based on the presence of larger/rhabdoid cells in a tumor, with an otherwise epithelioid morphology, exhibiting a polyphenotypic expression with mesenchymal markers (vimentin, CD34); a myogenic marker (desmin) along with epithelial markers (CK, CK7, EMA). There have been infrequent studies and case reports on this tumor [4,8-10]. Ultrastructurally, the rhabdoid cells noted in this tumor have been found to be displaying paranuclear, well-delineated filamentous aggregates, indenting the nucleus eccentrically along with tonofilaments around the cytoplasmic inclusion that in itself is a result of the filamentous aggregates [4]. In view of lack of fresh tissue, we could not perform an ultrastructural analysis. Nevertheless, the distinct cytoplasmic inclusions were well noted on conventional staining. The 'rhabdoid' histology has been seen in carcinomas and melanomas [11]. In cases of proximal-type ES, it forms an intrinsic part of the tumor morphology. However, its prognostic significance remains unclear [8]. Another close differential, which was entertained, was the rhabdoid tumor itself. In terms of its biological behavior, like its renal counterpart, an extra-renal rhabdoid tumor (ER RT) is quite aggressive and lethal. However, it is commonly known to occur in younger children. An ER RT shows inactivating mutations/deletions of both the alleles of tumor suppresser genes *hSNF5/IN11 *on chromosome 22q11.2 [12]. Similar deletion has also been noted in proximal-type ES [13]. It has been proposed that considering the aberrations on chromosome 22 as an epiphenomenon, proximal-type ES might be actually representing a form of a "complex" rhabdoid tumor [12]. There have been few studies dealing with the genetics of this subtype of ES, including a recent case report by Lee et al [14], who reported chromosomal gains of 5q32-qter, 12q24qter, and 22q by comparative genomic hybridization in this tumor.

The importance of identifying this subtype lies in its aggressive behavior was seen in our patient who, despite adjuvant CT/RT did not get rid of the local lesion. Although, fortunately he did not develop any metastatic lesions in view of the adjuvant treatment, the persistence of the tumor suggests a possibility of unclear margins of the first excision that was performed elsewhere.

Certain prognostic factors, including tumor size have been recognized in proximal-type ES [8]. Current accumulated data indicate that the presence of rhabdoid features in malignant tumors is related to an aggressive behavior, multimodal therapy resistance, a rapidly fatal outcome. At the same time it has been postulated that whether this grim prognosis is related to rhabdoid vs non-rhabdoid cell ratio with in the tumor is unclear [11,15,16]. In our case, the recurrent mass revealed an increase in the number of larger cells. It would be worthwhile to identify more of such cases clinical outcomes. Further, genetic studies would be helpful in establishing insights into the molecular events responsible for the polyphenotypic expression of this tumor with an uncertain differentiation.

## Conclusion

The importance of identifying this tumor relates to its aggressive behavior. A range of differentials needs to be kept and ruled out on the basis of the clinical profile, morphology and a wide panel of relevant IHC markers. A timely diagnosis, optimal treatment in form of a wide-excision with clear margins and a close-follow up can be helpful in anticipating recurrences and a fatal outcome that can be seen with this aggressive tumor.

## Conflict of interest

The author(s) declare that they have no competing interests.

## Authors' contributions

BR: Involved in the diagnosis of the case; design, preparation and drafting of the manuscript.

BDG: Collected references, clinical and follow-up details.

RFC: Overall supervision and has given the final approval of the manuscript.
